# Pyriform Aperture Enlargement Through a Rhinoplasty Approach

**DOI:** 10.1093/asjof/ojae115

**Published:** 2024-11-23

**Authors:** Anil R Shah, David P Grande, Hallie Buckner, Rachel Nordgren

## Abstract

**Background:**

The pyriform aperture can limit airflow in some patients. In this study, the authors describe a new surgical technique to access and modify the pyriform aperture.

**Objectives:**

Describe the technique to measure the width of the pyriform aperture and surgically enlarge the pyriform aperture using ultrasonic instrumentation. Quantify improvement in nasal airway function with pyriform aperture size and Nasal Obstruction Symptom Evaluation (NOSE) scores.

**Methods:**

The pyriform aperture was measured intraoperatively using calipers. Preoperative and postoperative NOSE scores were compared in a retrospective analysis to examine the effects of pyriform aperture enlargement on nasal breathing.

**Results:**

On average, pyriform aperture width was enlarged by 2.9 mm (*P* < .0001) and NOSE scores decreased from 69.4 to 8.3 at 6 months (*P* < .0001).

**Conclusions:**

The pyriform aperture can be modified through an open rhinoplasty approach, and enlargement of the pyriform aperture can improve nasal obstruction symptoms.

**Level of Evidence: 5:**

(Therapeutic) 
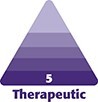

Rhinoplasty has been predominantly employed for aesthetics often at the expense of the stability and framework of the nasal airway. Guyuron described how lateral nasal bone osteotomies can impair nasal function by narrowing the nasal airway and compared the results for both low-to-low and high-to-low osteotomies.^1^ Although a high-to-low osteotomy had a lesser impact on nasal function, both osteotomy types resulted in some degree of functional decline because of nasal airway narrowing. Despite this, functional modifications during rhinoplasty can be used to enhance nasal airflow and improve a patient's quality of life. Our goal was to provide aesthetic narrowing of the nose, while also improving function.

Multiple etiologies of nasal obstruction exist, including septal deviation, turbinate hypertrophy, and narrow external and internal nasal valves. Improvements in nasal airflow are often limited to compensatory maneuvers at these sites, and the term “functional rhinoplasty” is typically associated with a septoplasty, turbinate reduction, and techniques to strengthen and support the cartilaginous structures of the nose. However, the nasal cartilages are mostly confined by the maxillary bone, which forms a border along the nasal airway, termed the “pyriform aperture.”

Roy et al^2^ described the bony nasal valve as an alternate site for nasal breathing impairment. Anatomically, the bony nasal valve is limited by the pyriform aperture, and it is defined by the following borders: bony nasal septum medially, head of the inferior turbinate inferiorly, and the nasal process of the maxilla laterally. Researchers examining nasal airflow physiology in their studies have demonstrated that a significant amount of nasal airway resistance is found in the region of the bony nasal valve or pyriform aperture.^3-5^

The pyriform aperture is the narrowest bony aspect of the nasal airway and small changes in its diameter can impact nasal airway resistance.^6,7^ However, few authors discuss reshaping the pyriform aperture to improve nasal breathing during nasal surgery. A proposed limitation to pyriform aperture enlargement has been gaining access to this bony region. The purpose of the authors of this study is to describe how the senior author uses a rhinoplasty approach to both access and enlarge the pyriform aperture to improve nasal airflow. We also describe a novel technique to assess and measure the width of the pyriform aperture.

## METHODS

### Patient Population and Data Collection

A retrospective analysis was performed on patients who underwent pyriform aperture enlargement in conjunction with a rhinoplasty procedure between October 2022 and April 2023. This project was approved by the Solutions Institutional Review Board. All surgical procedures were performed by the senior author. Patients completed a Nasal Obstruction Symptom Evaluation (NOSE) survey at their preoperative visit and at their 6-month follow-up visit. The NOSE survey is a validated tool used to assess and quantify nasal obstruction.^8^ Scores range from 0 through 100, with higher scores indicating worse nasal obstruction. Patients with a preoperative NOSE score ≥50 were included. Patients were excluded if their preoperative NOSE score was <50, because we find that patients often underreport nasal obstructive symptoms when undergoing a cosmetic rhinoplasty. In addition, patients who underwent a manipulation of the inferior turbinate, including submucosal reduction or outfracture, were excluded. The width of the pyriform aperture was measured before and after surgical intervention. A total of 49 patients were identified for the analysis. All patients had a septoplasty and spreader grafts performed in conjunction with pyriform aperture enlargement.

### Description of the Technique

In all patients, a cosmetic rhinoplasty was performed in conjunction with pyriform aperture enlargement. An open rhinoplasty approach was used to provide exposure to the pyriform aperture. The nose is opened with a V-shaped incision at the base of the columella. A supra-perichondrial approach is then used to lift the soft tissues from the lower lateral cartilages and the upper lateral cartilages. After the nasal bones are exposed, a subperiosteal dissection is initially performed along the dorsal aspect of the nasal bones. The periosteum is then swept laterally along the ascending process of the maxilla, degloving the nasal bones and maxilla from the overlying periosteum and soft tissue. Prior to surgical manipulation, the width of the pyriform aperture is measured using nasal calipers. The lateral border of the pyriform aperture is most easily identified by the bony region superior to the inferior turbinate, corresponding with the ascending process of the maxilla.

**Figure 1.**
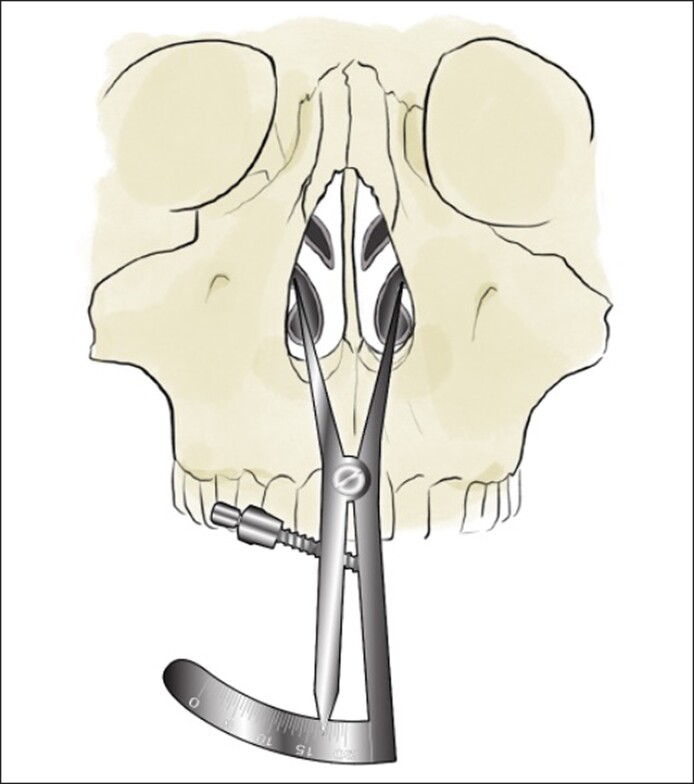
Illustration depicting pyriform aperture measurement using calipers prior to surgical enlargement.

**Figure 2.**
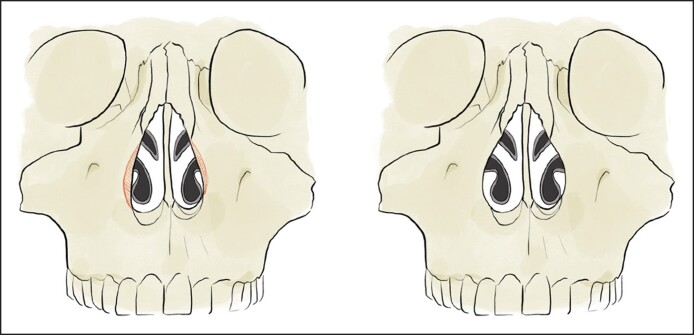
Illustration depicting the bony region to be removed outlined.

**Figure 3.**
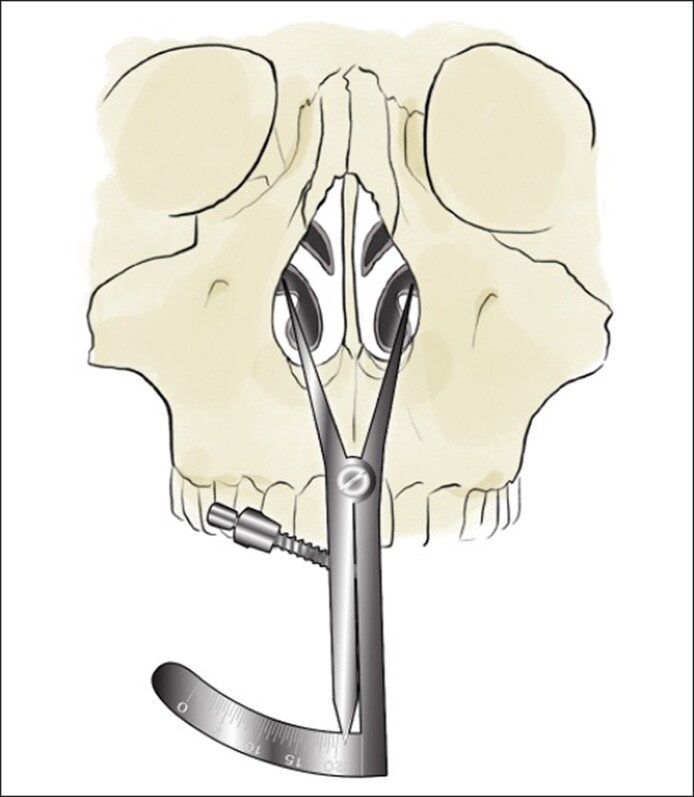
Illustration depicting measurement of the pyriform aperture after surgical enlargement.

One end of the caliper will be placed at this region and the other end will be placed at the same region on the contralateral side to measure the width of the pyriform aperture (Figure 1). The calipers should be placed firmly against the maxilla to compensate for any anesthetic or surgical edema. We then proceed with surgical manipulation of the pyriform aperture. In all patients, medial, transverse, and lateral osteotomies were performed using ultrasonic instrumentation. Under direct visualization, ultrasonic instrumentation is then used to reshape the maxilla and widen the pyriform aperture. The ascending process of the maxilla, which forms the lateral boundary of the bony nasal valve, is enlarged (Figure 2). This portion is palpable and often requires inferior dissection to identify the inferior, medial aspect of the pyriform aperture. The width of the pyriform aperture is then remeasured (Figure 3, Video).

### Statistical Analysis

A paired *t* test was used to compare the preoperative and postoperative NOSE scores and pyriform aperture measurements.

## RESULTS

**Figure 4.**
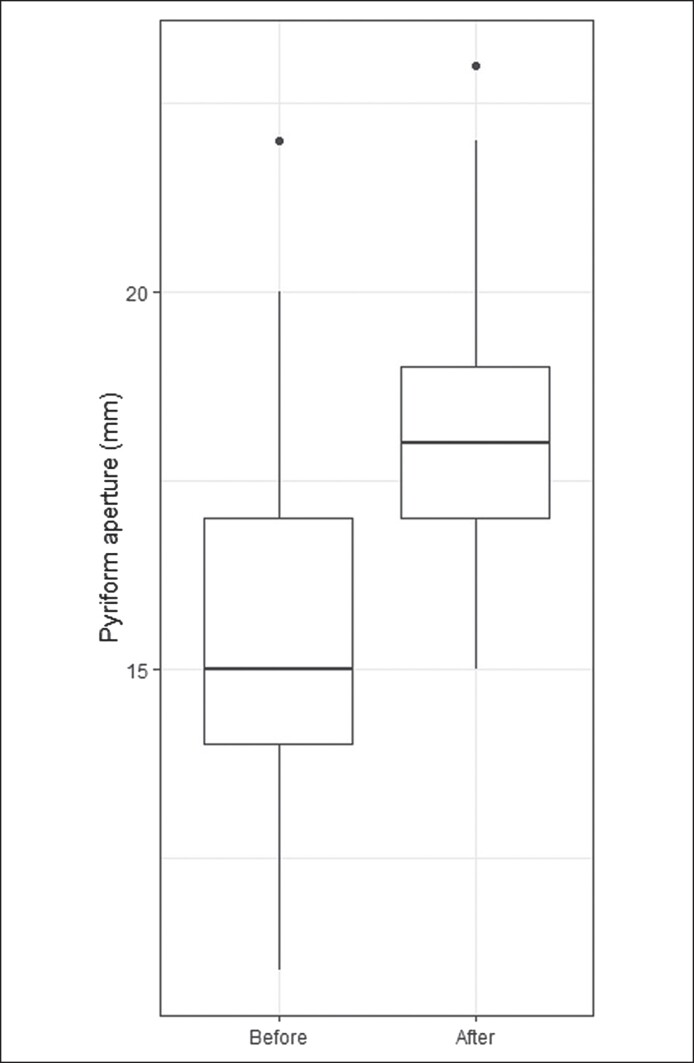
Preoperative and postoperative pyriform aperture width in millimeters. The boxes extend from the lower quartile to the upper quartile.

**Figure 5.**
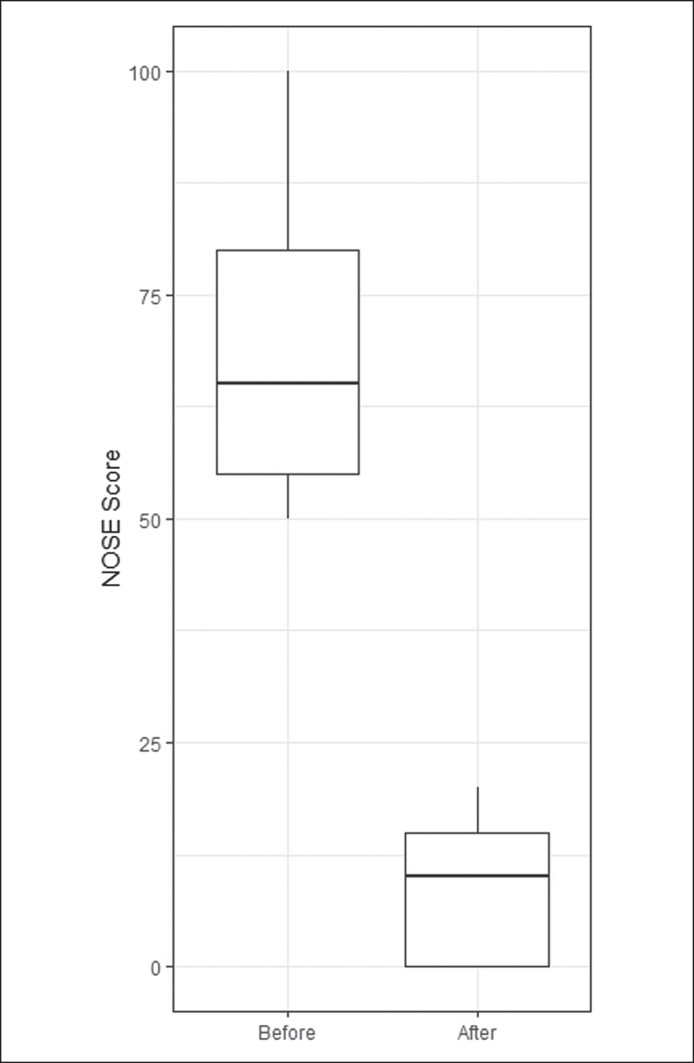
Preoperative and postoperative Nasal Obstruction Symptom Evaluation scores. The boxes extend from the lower quartile to the upper quartile.

A total of 49 patients who underwent cosmetic rhinoplasty with pyriform aperture enlargement were included in the analysis. Thirteen of the patients underwent revision rhinoplasty. The results are presented in Table 1. The pyriform aperture was enlarged in all patients, with a significant average increase of 2.9 mm (*P* < .0001; Figure 4). The NOSE scores decreased from a preoperative score of 69.4 to a postoperative score of 8.3 at 6 months (*P* < .0001; Figure 5).

**Table 1.** The Aperture Measurement and NOSE Score of Patients Who Underwent Cosmetic Rhinoplasty

*n* = 49	Mean (SD) Median (Q1, Q3)
Aperture before	15.3 (2.3) 15.0 (14.0, 17.0)
Aperture after	18.1 (2.0) 18.0 (17.0, 19.0)
Aperture difference	2.9 (1.0) 3.0 (2.0, 3.0)
NOSE before	69.4 (15.3) 65.0 (55.0, 80.0)
NOSE after	8.3 (6.4) 10.0 (0.0, 15.0)
NOSE difference	−61.1 (15.1) −60.0 (−70.0, −50.0)

NOSE, Nasal Obstruction Symptom Evaluation; SD, standard deviation.

There were no complications associated with pyriform aperture enlargement, including postoperative epistaxis, cosmetic deformity, nasal synechiae, septal perforation, palatal fistula, or sensory disturbances. All patients underwent septoplasty and spreader graft placement.

## DISCUSSION

This is the first paper in which the authors describe pyriform aperture enlargement through an open rhinoplasty approach. In our study, pyriform aperture enlargement resulted in an improvement in nasal obstructive symptoms based on a reduction in NOSE scores. The pyriform aperture forms the bony framework of the anterior nasal cavity and is a site of significant nasal airway resistance.^4^ It is bordered laterally by the nasal process of the maxilla, inferiorly by the horizontal process of the maxilla, and superiorly by the nasal bones. Increasing the size of the pyriform aperture has been shown to lead to an increase in nasal airflow and reduction in nasal obstructive symptoms.^2,9^ Computational fluid dynamics analyzing nasal airflow revealed an 8-fold increase in nasal airway resistance among children with congenital pyriform aperture stenosis.^10^ However, few researchers discuss reshaping and enlarging the pyriform aperture to improve nasal breathing in adult patients.

Douglas was the first to describe the resection of pyriform aperture through a sublabial approach in order to treat nasal obstruction.^11^ Woodhead then hypothesized that widening the pyriform aperture may improve lateral nasal wall collapse and described a technique using an endonasal Z-plasty incision.^12^ Other surgeons have further endorsed the use of sublabial incisions and various endonasal incisions to access and enlarge the pyriform aperture, with most patients reporting functional improvement after pyriform aperture enlargement.^2,9,13-17^

Our approach offers many advantages compared with the previously described techniques using sublabial or endonasal incisions. First, no additional incisions are required to access the pyriform aperture. This allows for less complications associated with additional incisions, including bleeding, scarring, and edema. Second, the nasal bones and maxilla are often exposed during an ultrasonic open rhinoplasty, thereby facilitating quick access to the pyriform aperture with further dissection along the ascending process of the maxilla. Third, the use of ultrasonic instrumentation allows for the rapid removal of bone without compromising precision. This device also allows for minimal soft tissue disruption, resulting in less intraoperative bleeding. The senior author has found that this approach is efficient and can be performed in under 10 min. There were also no complications or deleterious changes associated with the procedure. Results were sustained as evidenced by an improvement in NOSE scores at the 6-month follow-up.

Notably, the senior author did not perform a turbinate reduction technique in any of our patients, because this is primarily a compensatory maneuver performed during a rhinoplasty. The turbinate is a vital structure that humidifies and regulates air temperature, and its presence is important in the normal sensation of nasal breathing. We find that widening the pyriform aperture avoids the need for any manipulation of the inferior turbinate.

A limitation of this study is that the patients underwent various other functional and cosmetic modifications to the nose in addition to pyriform aperture enlargement. This includes septoplasty, spreader grafts, tip reshaping, and osteotomies. Therefore, improvement solely attributed to enlarging the pyriform aperture cannot be determined.

The authors of this study demonstrate that pyriform aperture enlargement appears to be safe and effective when performed in conjunction with rhinoplasty. This technique improves nasal breathing, and we propose that surgeons consider pyriform aperture enlargement during rhinoplasty, especially when maneuvers that narrow the airway are performed, such as lateral osteotomies. As our understanding of airway physiology improves with computational flow dynamics and patient outcomes, pyriform aperture enlargement may play a larger role in improving nasal function.

## CONCLUSIONS

Pyriform aperture enlargement can be successfully performed through an open rhinoplasty approach. This technique improves nasal breathing, and we propose that surgeons consider pyriform aperture enlargement during rhinoplasty, especially when maneuvers that narrow the airway are performed, such as lateral osteotomies.
